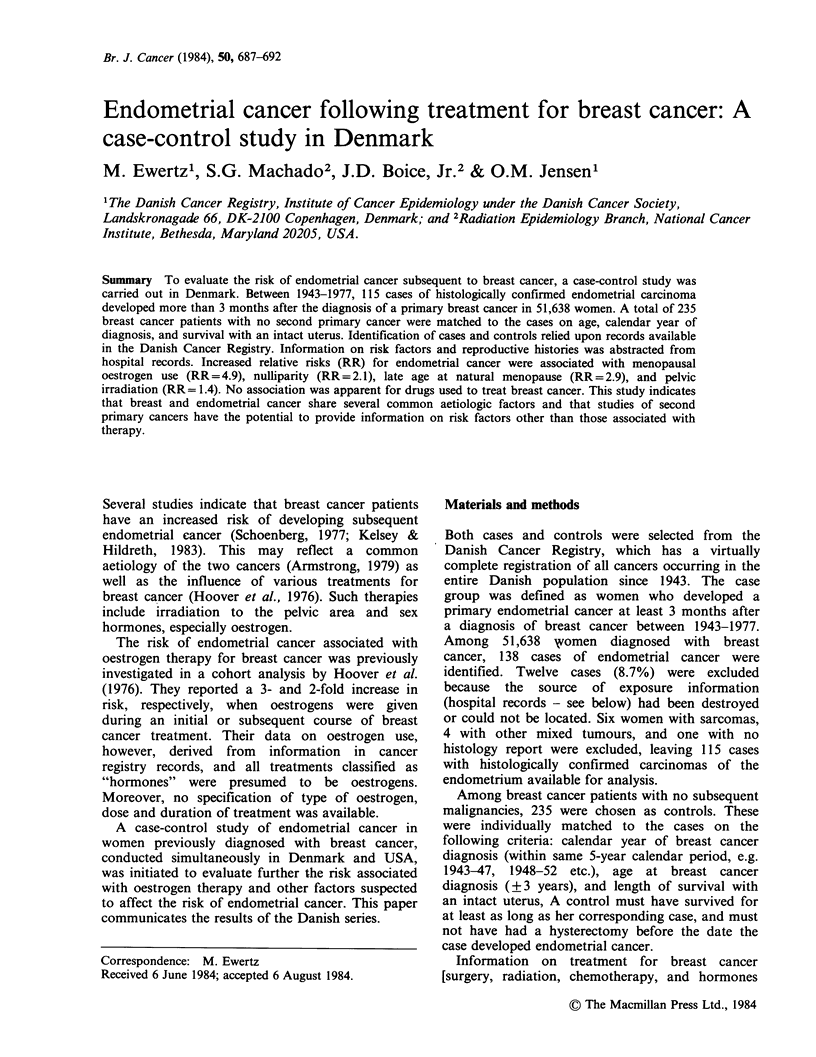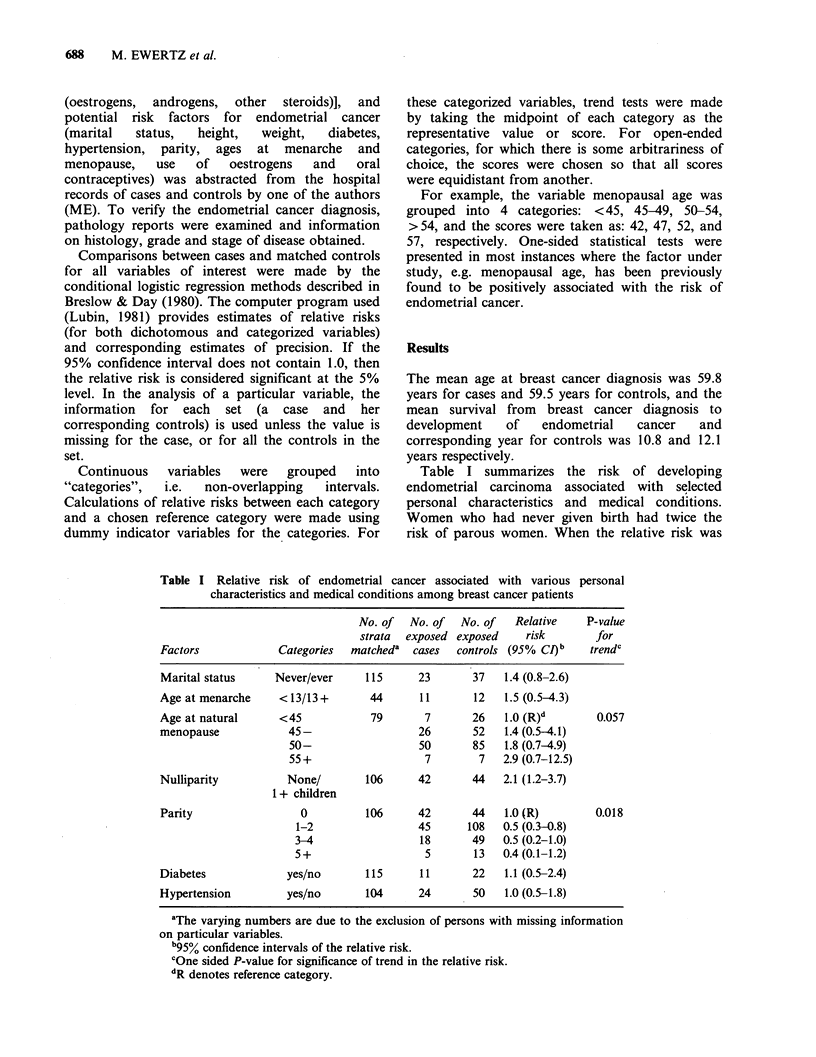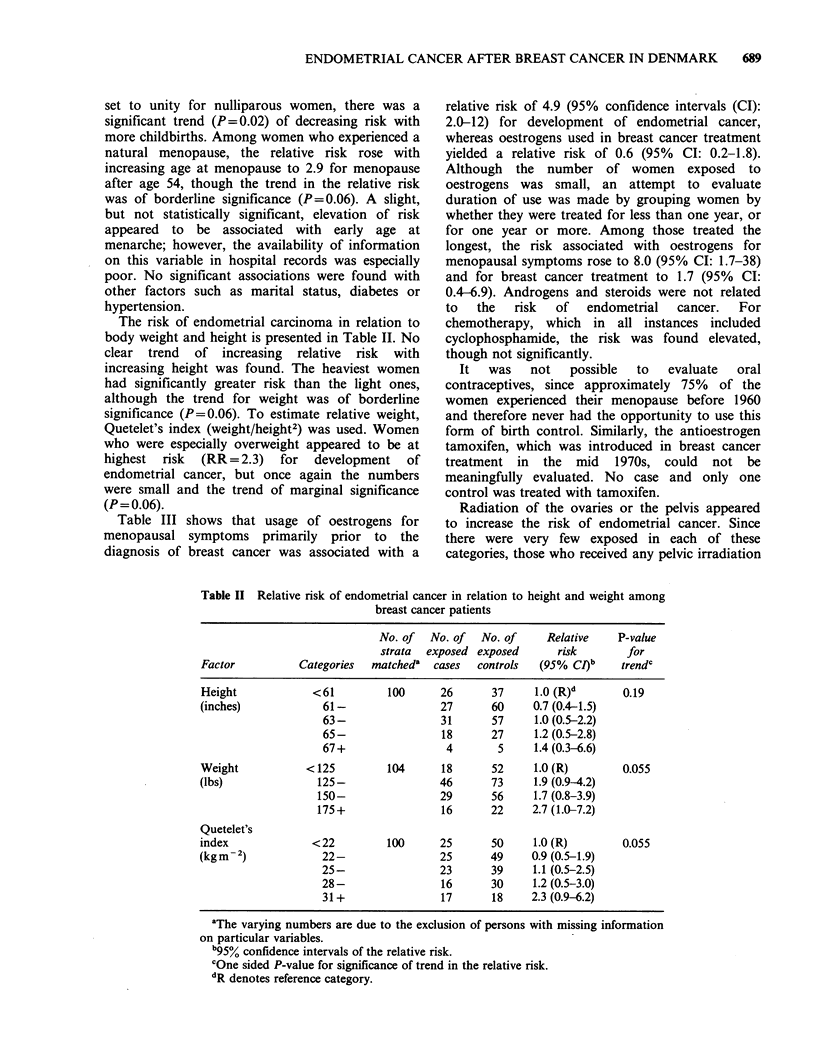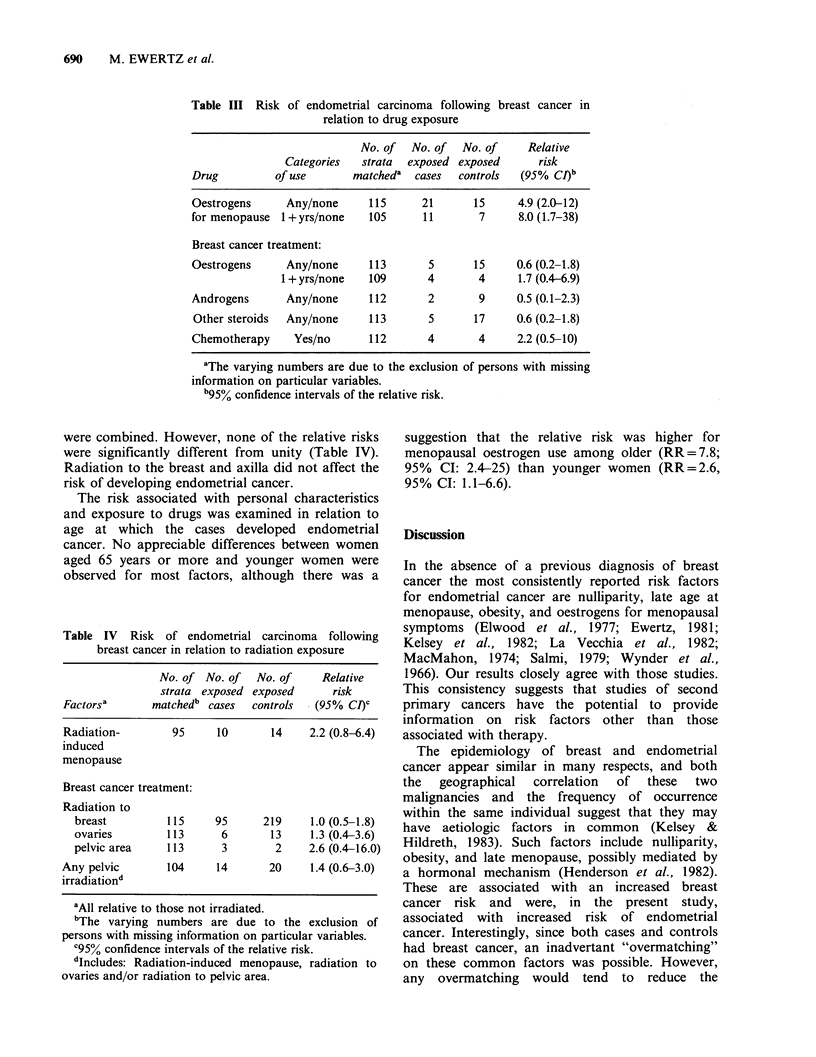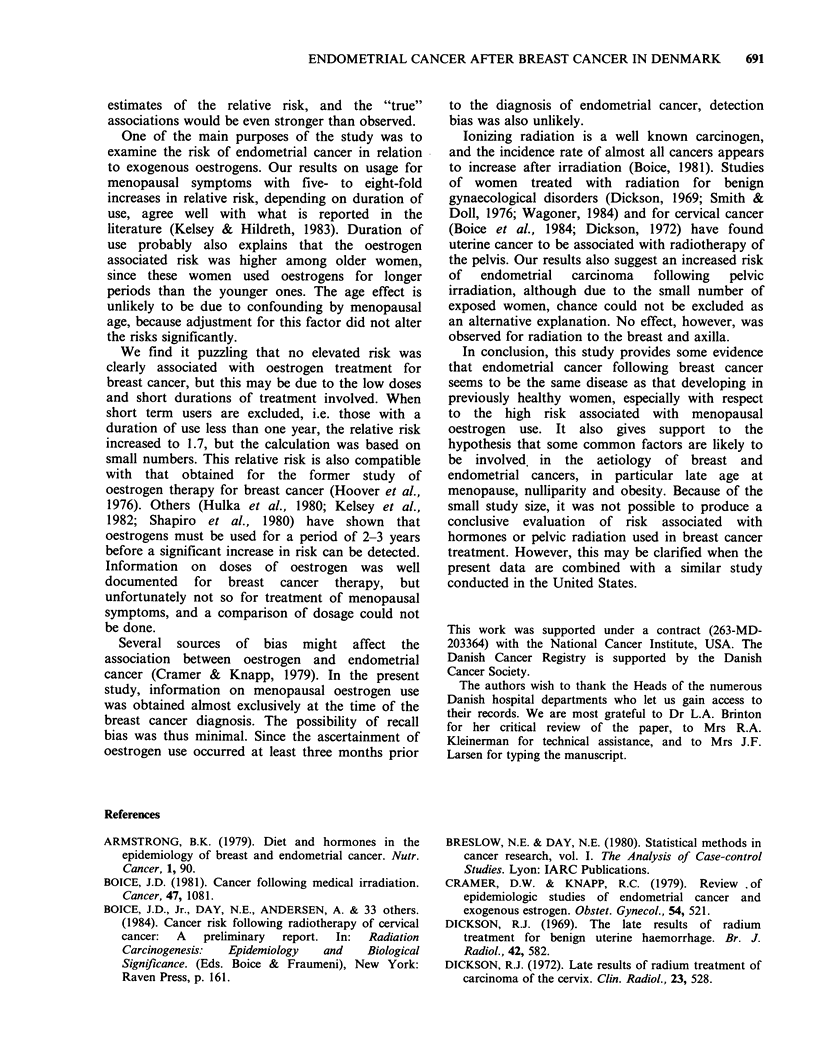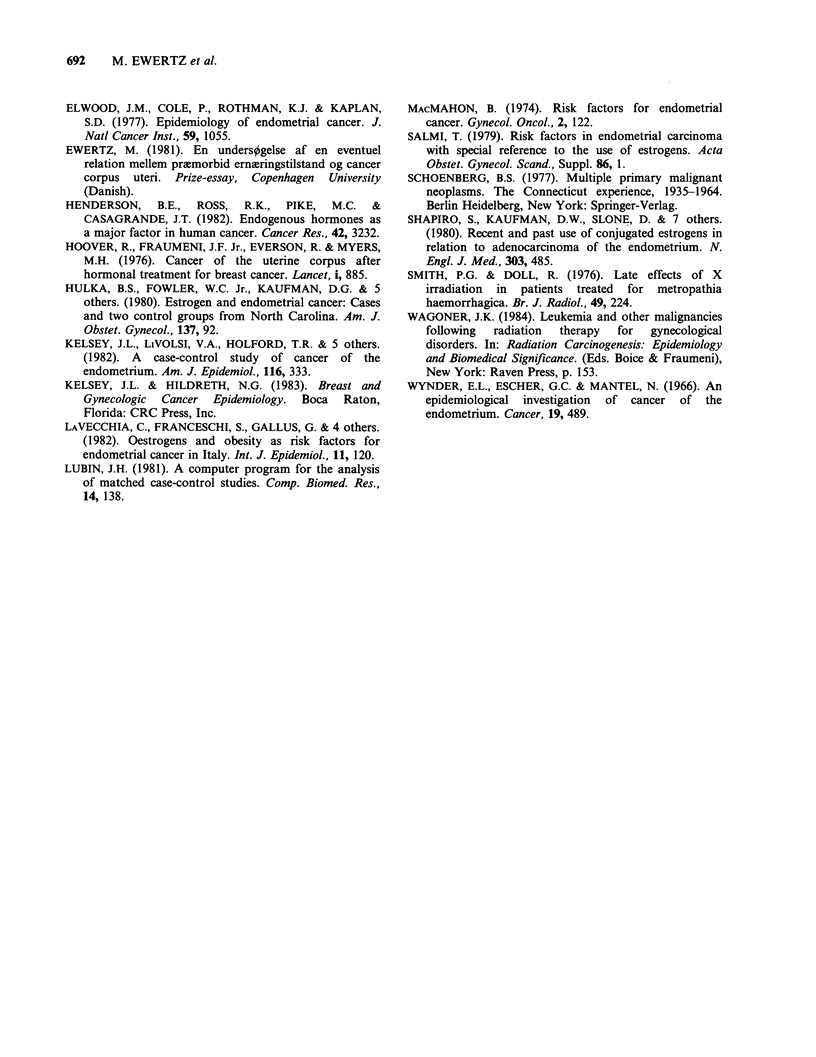# Endometrial cancer following treatment for breast cancer: a case-control study in Denmark.

**DOI:** 10.1038/bjc.1984.237

**Published:** 1984-11

**Authors:** M. Ewertz, S. G. Machado, J. D. Boice, O. M. Jensen

## Abstract

To evaluate the risk of endometrial cancer subsequent to breast cancer, a case-control study was carried out in Denmark. Between 1943-1977, 115 cases of histologically confirmed endometrial carcinoma developed more than 3 months after the diagnosis of a primary breast cancer in 51,638 women. A total of 235 breast cancer patients with no second primary cancer were matched to the cases on age, calendar year of diagnosis, and survival with an intact uterus. Identification of cases and controls relied upon records available in the Danish Cancer Registry. Information on risk factors and reproductive histories was abstracted from hospital records. Increased relative risks (RR) for endometrial cancer were associated with menopausal oestrogen use (RR = 4.9), nulliparity (RR = 2.1), late age at natural menopause (RR = 2.9), and pelvic irradiation (RR = 1.4). No association was apparent for drugs used to treat breast cancer. This study indicates that breast and endometrial cancer share several common aetiologic factors and that studies of second primary cancers have the potential to provide information on risk factors other than those associated with therapy.


*Br. J. Cancer* (1984), **50**, 687–692

### Endometrial cancer following treatment for breast cancer: A case-control study in Denmark

M. Ewertz[1], S.G. Machado[2], J.D. Boice, Jr.[2] & O.M. Jensen[1]

[1]*The Danish Cancer Registry, Institute of Cancer Epidemiology under the Danish Cancer Society, Landskronagade 66, DK-2100 Copenhagen, Denmark; and* [2]*Radiation Epidemiology Branch, National Cancer Institute, Bethesda, Maryland 20205, USA.*

**Summary** To evaluate the risk of endometrial cancer subsequent to breast cancer, a case-control study was carried out in Denmark. Between 1943–1977, 115 cases of histologically confirmed endometrial carcinoma developed more than 3 months after the diagnosis of a primary breast cancer in 51,638 women. A total of 235 breast cancer patients with no second primary cancer were matched to the cases on age, calendar year of diagnosis, and survival with an intact uterus. Identification of cases and controls relied upon records available in the Danish Cancer Registry. Information on risk factors and reproductive histories was abstracted from hospital records. Increased relative risks (RR) for endometrial cancer were associated with menopausal oestrogen use (RR=4.9), nulliparity (RR=2.1), late age at natural menopause (RR=2.9), and pelvic irradiation (RR=1.4). No association was apparent for drugs used to treat breast cancer. This study indicates that breast and endometrial cancer share several common aetiologic factors and that studies of second primary cancers have the potential to provide information on risk factors other than those associated with therapy.

Several studies indicate that breast cancer patients have an increased risk of developing subsequent endometrial cancer (Schoenberg, 1977; Kelsey & Hildreth, 1983). This may reflect a common aetiology of the two cancers (Armstrong, 1979) as well as the influence of various treatments for breast cancer (Hoover *et al.*, 1976). Such therapies include irradiation to the pelvic area and sex hormones, especially oestrogen.

The risk of endometrial cancer associated with oestrogen therapy for breast cancer was previously investigated in a cohort analysis by Hoover *et al.* (1976). They reported a 3- and 2-fold increase in risk, respectively, when oestrogens were given during an initial or subsequent course of breast cancer treatment. Their data on oestrogen use, however, derived from information in cancer registry records, and all treatments classified as "hormones" were presumed to be oestrogens. Moreover, no specification of type of oestrogen, dose and duration of treatment was available.

A case-control study of endometrial cancer in women previously diagnosed with breast cancer, conducted simultaneously in Denmark and USA, was initiated to evaluate further the risk associated with oestrogen therapy and other factors suspected to affect the risk of endometrial cancer. This paper communicates the results of the Danish series.

Correspondence: M. Ewertz
Received 6 June 1984; accepted 6 August 1984.

### Materials and methods

Both cases and controls were selected from the Danish Cancer Registry, which has a virtually complete registration of all cancers occurring in the entire Danish population since 1943. The case group was defined as women who developed a primary endometrial cancer at least 3 months after a diagnosis of breast cancer between 1943–1977. Among 51,638 women diagnosed with breast cancer, 138 cases of endometrial cancer were identified. Twelve cases (8.7%) were excluded because the source of exposure information (hospital records – see below) had been destroyed or could not be located. Six women with sarcomas, 4 with other mixed tumours, and one with no histology report were excluded, leaving 115 cases with histologically confirmed carcinomas of the endometrium available for analysis.

Among breast cancer patients with no subsequent malignancies, 235 were chosen as controls. These were individually matched to the cases on the following criteria: calendar year of breast cancer diagnosis (within same 5-year calendar period, e.g. 1943–47, 1948–52 etc.), age at breast cancer diagnosis ($\pm 3$ years), and length of survival with an intact uterus, A control must have survived for at least as long as her corresponding case, and must not have had a hysterectomy before the date the case developed endometrial cancer.

Information on treatment for breast cancer [surgery, radiation, chemotherapy, and hormones

© The Macmillan Press Ltd., 1984

(oestrogens, androgens, other steroids)], and potential risk factors for endometrial cancer (marital status, height, weight, diabetes, hypertension, parity, ages at menarche and menopause, use of oestrogens and oral contraceptives) was abstracted from the hospital records of cases and controls by one of the authors (ME). To verify the endometrial cancer diagnosis, pathology reports were examined and information on histology, grade and stage of disease obtained.

Comparisons between cases and matched controls for all variables of interest were made by the conditional logistic regression methods described in Breslow & Day (1980). The computer program used (Lubin, 1981) provides estimates of relative risks (for both dichotomous and categorized variables) and corresponding estimates of precision. If the 95% confidence interval does not contain 1.0, then the relative risk is considered significant at the 5% level. In the analysis of a particular variable, the information for each set (a case and her corresponding controls) is used unless the value is missing for the case, or for all the controls in the set.

Continuous variables were grouped into "categories", i.e. non-overlapping intervals. Calculations of relative risks between each category and a chosen reference category were made using dummy indicator variables for the categories. For these categorized variables, trend tests were made by taking the midpoint of each category as the representative value or score. For open-ended categories, for which there is some arbitrariness of choice, the scores were chosen so that all scores were equidistant from another.

For example, the variable menopausal age was grouped into 4 categories: <45, 45–49, 50–54, >54, and the scores were taken as: 42, 47, 52, and 57, respectively. One-sided statistical tests were presented in most instances where the factor under study, e.g. menopausal age, has been previously found to be positively associated with the risk of endometrial cancer.

### Results

The mean age at breast cancer diagnosis was 59.8 years for cases and 59.5 years for controls, and the mean survival from breast cancer diagnosis to development of endometrial cancer and corresponding year for controls was 10.8 and 12.1 years respectively.

Table I summarizes the risk of developing endometrial carcinoma associated with selected personal characteristics and medical conditions. Women who had never given birth had twice the risk of parous women. When the relative risk was

**Table I** Relative risk of endometrial cancer associated with various personal characteristics and medical conditions among breast cancer patients

| *Factors* | *Categories* | *No. of strata matched*[a] | *No. of exposed cases* | *No. of exposed controls* | *Relative risk (95% CI)*[b] | P-*value for trend*[c] |
|---|---|---|---|---|---|---|
| Marital status | Never/ever | 115 | 23 | 37 | 1.4 (0.8–2.6) | |
| Age at menarche | <13/13+ | 44 | 11 | 12 | 1.5 (0.5–4.3) | |
| Age at natural menopause | <45 | 79 | 7 | 26 | 1.0 (R)[d] | 0.057 |
| | 45– | | 26 | 52 | 1.4 (0.5–4.1) | |
| | 50– | | 50 | 85 | 1.8 (0.7–4.9) | |
| | 55+ | | 7 | 7 | 2.9 (0.7–12.5) | |
| Nulliparity | None/ 1+ children | 106 | 42 | 44 | 2.1 (1.2–3.7) | |
| Parity | 0 | 106 | 42 | 44 | 1.0 (R) | 0.018 |
| | 1–2 | | 45 | 108 | 0.5 (0.3–0.8) | |
| | 3–4 | | 18 | 49 | 0.5 (0.2–1.0) | |
| | 5+ | | 5 | 13 | 0.4 (0.1–1.2) | |
| Diabetes | yes/no | 115 | 11 | 22 | 1.1 (0.5–2.4) | |
| Hypertension | yes/no | 104 | 24 | 50 | 1.0 (0.5–1.8) | |

[a]The varying numbers are due to the exclusion of persons with missing information on particular variables.
[b]95% confidence intervals of the relative risk.
[c]One sided *P*-value for significance of trend in the relative risk.
[d]R denotes reference category.

set to unity for nulliparous women, there was a significant trend ($P = 0.02$) of decreasing risk with more childbirths. Among women who experienced a natural menopause, the relative risk rose with increasing age at menopause to 2.9 for menopause after age 54, though the trend in the relative risk was of borderline significance ($P = 0.06$). A slight, but not statistically significant, elevation of risk appeared to be associated with early age at menarche; however, the availability of information on this variable in hospital records was especially poor. No significant associations were found with other factors such as marital status, diabetes or hypertension.

The risk of endometrial carcinoma in relation to body weight and height is presented in Table II. No clear trend of increasing relative risk with increasing height was found. The heaviest women had significantly greater risk than the light ones, although the trend for weight was of borderline significance ($P = 0.06$). To estimate relative weight, Quetelet's index (weight/height$^2$) was used. Women who were especially overweight appeared to be at highest risk (RR = 2.3) for development of endometrial cancer, but once again the numbers were small and the trend of marginal significance ($P = 0.06$).

Table III shows that usage of oestrogens for menopausal symptoms primarily prior to the diagnosis of breast cancer was associated with a relative risk of 4.9 (95% confidence intervals (CI): 2.0–12) for development of endometrial cancer, whereas oestrogens used in breast cancer treatment yielded a relative risk of 0.6 (95% CI: 0.2–1.8). Although the number of women exposed to oestrogens was small, an attempt to evaluate duration of use was made by grouping women by whether they were treated for less than one year, or for one year or more. Among those treated the longest, the risk associated with oestrogens for menopausal symptoms rose to 8.0 (95% CI: 1.7–38) and for breast cancer treatment to 1.7 (95% CI: 0.4–6.9). Androgens and steroids were not related to the risk of endometrial cancer. For chemotherapy, which in all instances included cyclophosphamide, the risk was found elevated, though not significantly.

It was not possible to evaluate oral contraceptives, since approximately 75% of the women experienced their menopause before 1960 and therefore never had the opportunity to use this form of birth control. Similarly, the antioestrogen tamoxifen, which was introduced in breast cancer treatment in the mid 1970s, could not be meaningfully evaluated. No case and only one control was treated with tamoxifen.

Radiation of the ovaries or the pelvis appeared to increase the risk of endometrial cancer. Since there were very few exposed in each of these categories, those who received any pelvic irradiation

**Table II** Relative risk of endometrial cancer in relation to height and weight among breast cancer patients

| *Factor* | *Categories* | *No. of strata matched*[a] | *No. of exposed cases* | *No. of exposed controls* | *Relative risk (95% CI)*[b] | P-*value for trend*[c] |
|---|---|---|---|---|---|---|
| Height (inches) | <61 | 100 | 26 | 37 | 1.0 (R)[d] | 0.19 |
| | 61– | | 27 | 60 | 0.7 (0.4–1.5) | |
| | 63– | | 31 | 57 | 1.0 (0.5–2.2) | |
| | 65– | | 18 | 27 | 1.2 (0.5–2.8) | |
| | 67+ | | 4 | 5 | 1.4 (0.3–6.6) | |
| Weight (lbs) | <125 | 104 | 18 | 52 | 1.0 (R) | 0.055 |
| | 125– | | 46 | 73 | 1.9 (0.9–4.2) | |
| | 150– | | 29 | 56 | 1.7 (0.8–3.9) | |
| | 175+ | | 16 | 22 | 2.7 (1.0–7.2) | |
| Quetelet's index ($kg\,m^{-2}$) | <22 | 100 | 25 | 50 | 1.0 (R) | 0.055 |
| | 22– | | 25 | 49 | 0.9 (0.5–1.9) | |
| | 25– | | 23 | 39 | 1.1 (0.5–2.5) | |
| | 28– | | 16 | 30 | 1.2 (0.5–3.0) | |
| | 31+ | | 17 | 18 | 2.3 (0.9–6.2) | |

[a]The varying numbers are due to the exclusion of persons with missing information on particular variables.
[b]95% confidence intervals of the relative risk.
[c]One sided $P$-value for significance of trend in the relative risk.
[d]R denotes reference category.

**Table III** Risk of endometrial carcinoma following breast cancer in relation to drug exposure

| *Drug* | *Categories of use* | *No. of strata matched*[a] | *No. of exposed cases* | *No. of exposed controls* | *Relative risk (95% CI)*[b] |
|---|---|---|---|---|---|
| Oestrogens | Any/none | 115 | 21 | 15 | 4.9 (2.0–12) |
| for menopause | 1 + yrs/none | 105 | 11 | 7 | 8.0 (1.7–38) |
| Breast cancer treatment: | | | | | |
| Oestrogens | Any/none | 113 | 5 | 15 | 0.6 (0.2–1.8) |
| | 1 + yrs/none | 109 | 4 | 4 | 1.7 (0.4–6.9) |
| Androgens | Any/none | 112 | 2 | 9 | 0.5 (0.1–2.3) |
| Other steroids | Any/none | 113 | 5 | 17 | 0.6 (0.2–1.8) |
| Chemotherapy | Yes/no | 112 | 4 | 4 | 2.2 (0.5–10) |

[a]The varying numbers are due to the exclusion of persons with missing information on particular variables.
[b]95% confidence intervals of the relative risk.

were combined. However, none of the relative risks were significantly different from unity (Table IV). Radiation to the breast and axilla did not affect the risk of developing endometrial cancer.

The risk associated with personal characteristics and exposure to drugs was examined in relation to age at which the cases developed endometrial cancer. No appreciable differences between women aged 65 years or more and younger women were observed for most factors, although there was a suggestion that the relative risk was higher for menopausal oestrogen use among older (RR = 7.8; 95% CI: 2.4–25) than younger women (RR = 2.6, 95% CI: 1.1–6.6).

**Table IV** Risk of endometrial carcinoma following breast cancer in relation to radiation exposure

| *Factors*[a] | *No. of strata matched*[b] | *No. of exposed cases* | *No. of exposed controls* | *Relative risk (95% CI)*[c] |
|---|---|---|---|---|
| Radiation-induced menopause | 95 | 10 | 14 | 2.2 (0.8–6.4) |
| Breast cancer treatment: | | | | |
| Radiation to | | | | |
| breast | 115 | 95 | 219 | 1.0 (0.5–1.8) |
| ovaries | 113 | 6 | 13 | 1.3 (0.4–3.6) |
| pelvic area | 113 | 3 | 2 | 2.6 (0.4–16.0) |
| Any pelvic irradiation[d] | 104 | 14 | 20 | 1.4 (0.6–3.0) |

[a]All relative to those not irradiated.
[b]The varying numbers are due to the exclusion of persons with missing information on particular variables.
[c]95% confidence intervals of the relative risk.
[d]Includes: Radiation-induced menopause, radiation to ovaries and/or radiation to pelvic area.

### Discussion

In the absence of a previous diagnosis of breast cancer the most consistently reported risk factors for endometrial cancer are nulliparity, late age at menopause, obesity, and oestrogens for menopausal symptoms (Elwood *et al.*, 1977; Ewertz, 1981; Kelsey *et al.*, 1982; La Vecchia *et al.*, 1982; MacMahon, 1974; Salmi, 1979; Wynder *et al.*, 1966). Our results closely agree with those studies. This consistency suggests that studies of second primary cancers have the potential to provide information on risk factors other than those associated with therapy.

The epidemiology of breast and endometrial cancer appear similar in many respects, and both the geographical correlation of these two malignancies and the frequency of occurrence within the same individual suggest that they may have aetiologic factors in common (Kelsey & Hildreth, 1983). Such factors include nulliparity, obesity, and late menopause, possibly mediated by a hormonal mechanism (Henderson *et al.*, 1982). These are associated with an increased breast cancer risk and were, in the present study, associated with increased risk of endometrial cancer. Interestingly, since both cases and controls had breast cancer, an inadvertant "overmatching" on these common factors was possible. However, any overmatching would tend to reduce the estimates of the relative risk, and the "true" associations would be even stronger than observed.

One of the main purposes of the study was to examine the risk of endometrial cancer in relation to exogenous oestrogens. Our results on usage for menopausal symptoms with five- to eight-fold increases in relative risk, depending on duration of use, agree well with what is reported in the literature (Kelsey & Hildreth, 1983). Duration of use probably also explains that the oestrogen associated risk was higher among older women, since these women used oestrogens for longer periods than the younger ones. The age effect is unlikely to be due to confounding by menopausal age, because adjustment for this factor did not alter the risks significantly.

We find it puzzling that no elevated risk was clearly associated with oestrogen treatment for breast cancer, but this may be due to the low doses and short durations of treatment involved. When short term users are excluded, i.e. those with a duration of use less than one year, the relative risk increased to 1.7, but the calculation was based on small numbers. This relative risk is also compatible with that obtained for the former study of oestrogen therapy for breast cancer (Hoover *et al.*, 1976). Others (Hulka *et al.*, 1980; Kelsey *et al.*, 1982; Shapiro *et al.*, 1980) have shown that oestrogens must be used for a period of 2–3 years before a significant increase in risk can be detected. Information on doses of oestrogen was well documented for breast cancer therapy, but unfortunately not so for treatment of menopausal symptoms, and a comparison of dosage could not be done.

Several sources of bias might affect the association between oestrogen and endometrial cancer (Cramer & Knapp, 1979). In the present study, information on menopausal oestrogen use was obtained almost exclusively at the time of the breast cancer diagnosis. The possibility of recall bias was thus minimal. Since the ascertainment of oestrogen use occurred at least three months prior to the diagnosis of endometrial cancer, detection bias was also unlikely.

Ionizing radiation is a well known carcinogen, and the incidence rate of almost all cancers appears to increase after irradiation (Boice, 1981). Studies of women treated with radiation for benign gynaecological disorders (Dickson, 1969; Smith & Doll, 1976; Wagoner, 1984) and for cervical cancer (Boice *et al.*, 1984; Dickson, 1972) have found uterine cancer to be associated with radiotherapy of the pelvis. Our results also suggest an increased risk of endometrial carcinoma following pelvic irradiation, although due to the small number of exposed women, chance could not be excluded as an alternative explanation. No effect, however, was observed for radiation to the breast and axilla.

In conclusion, this study provides some evidence that endometrial cancer following breast cancer seems to be the same disease as that developing in previously healthy women, especially with respect to the high risk associated with menopausal oestrogen use. It also gives support to the hypothesis that some common factors are likely to be involved in the aetiology of breast and endometrial cancers, in particular late age at menopause, nulliparity and obesity. Because of the small study size, it was not possible to produce a conclusive evaluation of risk associated with hormones or pelvic radiation used in breast cancer treatment. However, this may be clarified when the present data are combined with a similar study conducted in the United States.

This work was supported under a contract (263-MD-203364) with the National Cancer Institute, USA. The Danish Cancer Registry is supported by the Danish Cancer Society.

The authors wish to thank the Heads of the numerous Danish hospital departments who let us gain access to their records. We are most grateful to Dr L.A. Brinton for her critical review of the paper, to Mrs R.A. Kleinerman for technical assistance, and to Mrs J.F. Larsen for typing the manuscript.

**References**

ARMSTRONG, B.K. (1979). Diet and hormones in the epidemiology of breast and endometrial cancer. *Nutr. Cancer*, **1,** 90.

BOICE, J.D. (1981). Cancer following medical irradiation. *Cancer*, **47,** 1081.

BOICE, J.D., Jr., DAY, N.E., ANDERSEN, A. & 33 others. (1984). Cancer risk following radiotherapy of cervical cancer: A preliminary report. In: *Radiation Carcinogenesis: Epidemiology and Biological Significance*. (Eds. Boice & Fraumeni), New York: Raven Press, p. 161.

BRESLOW, N.E. & DAY, N.E. (1980). Statistical methods in cancer research, vol. I. *The Analysis of Case-control Studies*. Lyon: IARC Publications.

CRAMER, D.W. & KNAPP, R.C. (1979). Review of epidemiologic studies of endometrial cancer and exogenous estrogen. *Obstet. Gynecol.*, **54,** 521.

DICKSON, R.J. (1969). The late results of radium treatment for benign uterine haemorrhage. *Br. J. Radiol.*, **42,** 582.

DICKSON, R.J. (1972). Late results of radium treatment of carcinoma of the cervix. *Clin. Radiol.*, **23,** 528.

ELWOOD, J.M., COLE, P., ROTHMAN, K.J. & KAPLAN, S.D. (1977). Epidemiology of endometrial cancer. *J. Natl Cancer Inst.*, **59**, 1055.

EWERTZ, M. (1981). En undersøgelse af en eventuel relation mellem præmorbid ernæringstilstand og cancer corpus uteri. *Prize-essay, Copenhagen University* (Danish).

HENDERSON, B.E., ROSS, R.K., PIKE, M.C. & CASAGRANDE, J.T. (1982). Endogenous hormones as a major factor in human cancer. *Cancer Res.*, **42**, 3232.

HOOVER, R., FRAUMENI, J.F. Jr., EVERSON, R. & MYERS, M.H. (1976). Cancer of the uterine corpus after hormonal treatment for breast cancer. *Lancet*, **i**, 885.

HULKA, B.S., FOWLER, W.C. Jr., KAUFMAN, D.G. & 5 others. (1980). Estrogen and endometrial cancer: Cases and two control groups from North Carolina. *Am. J. Obstet. Gynecol.*, **137**, 92.

KELSEY, J.L., LIVOLSI, V.A., HOLFORD, T.R. & 5 others. (1982). A case-control study of cancer of the endometrium. *Am. J. Epidemiol.*, **116**, 333.

KELSEY, J.L. & HILDRETH, N.G. (1983). *Breast and Gynecologic Cancer Epidemiology*. Boca Raton, Florida: CRC Press, Inc.

LAVECCHIA, C., FRANCESCHI, S., GALLUS, G. & 4 others. (1982). Oestrogens and obesity as risk factors for endometrial cancer in Italy. *Int. J. Epidemiol.*, **11**, 120.

LUBIN, J.H. (1981). A computer program for the analysis of matched case-control studies. *Comp. Biomed. Res.*, **14**, 138.

MACMAHON, B. (1974). Risk factors for endometrial cancer. *Gynecol. Oncol.*, **2**, 122.

SALMI, T. (1979). Risk factors in endometrial carcinoma with special reference to the use of estrogens. *Acta Obstet. Gynecol. Scand.*, Suppl. **86**, 1.

SCHOENBERG, B.S. (1977). Multiple primary malignant neoplasms. The Connecticut experience, 1935–1964. Berlin Heidelberg, New York: Springer-Verlag.

SHAPIRO, S., KAUFMAN, D.W., SLONE, D. & 7 others. (1980). Recent and past use of conjugated estrogens in relation to adenocarcinoma of the endometrium. *N. Engl. J. Med.*, **303**, 485.

SMITH, P.G. & DOLL, R. (1976). Late effects of X irradiation in patients treated for metropathia haemorrhagica. *Br. J. Radiol.*, **49**, 224.

WAGONER, J.K. (1984). Leukemia and other malignancies following radiation therapy for gynecological disorders. In: *Radiation Carcinogenesis: Epidemiology and Biomedical Significance*. (Eds. Boice & Fraumeni), New York: Raven Press, p. 153.

WYNDER, E.L., ESCHER, G.C. & MANTEL, N. (1966). An epidemiological investigation of cancer of the endometrium. *Cancer*, **19**, 489.